# Supplementary material for: Are carbon emissions trading and green financial instruments synergistic? -Comprehensive quantitative research based on content analysis
Source: PLoS One. 2024 Mar 7;19(3):e0298601. doi: 10.1371/journal.pone.0298601 (PMC10919601; doi:10.1371/journal.pone.0298601)
Supplement: S1 Data — (ZIP) [file pone.0298601.s001.zip › Data/地级市Z_F工作报告.zip!/╜¡╦╒/╤∩╓▌╩╨/└┤╫╘╣1⁄2╓┌║┼í╛┬φ┐╦╩2╛▌═°í┐_2019.docx]

各位代表：

现在，我代表扬州市人民政府向大会报告工作，请予审议，并请市政协委员和其他列席人员提出意见。

2018年工作回顾

过去一年，我们坚持以习近平新时代中国特色社会主义思想为指导，全面贯彻党的十九大精神，深入落实习近平总书记对江苏重要指示要求，全面落实省委省政府决策部署，在市委正确领导下，践行新发展理念，把握稳中求进工作总基调，深化供给侧结构性改革，打好“三大攻坚战”，统筹做好改革发展稳定工作，全市高质量发展实现良好开局。

经济运行稳中向好。预计地区生产总值增长7%左右；一般公共预算收入340亿元，增长6.2%，其中税收占比80%，提升4.6个百分点；上汽大众仪征二期等一批百亿级重大项目成功落户，固定资产投资增长11%，增幅全省第一，其中制造业投资占比60%，提升2.6个百分点；社会消费品零售总额增长9.3%，增幅居全省前列；规上工业企业利润增长25%；高新技术产业产值占规上工业产值比重46%，提升1个百分点；绿色优质农产品占比35%；全社会研发投入增长10%，万人发明专利拥有量12件，同比增加2.2件。

城市形象大幅跃升。新建、提升各类城市公园65个，实施市区路口园艺化改造41处，完成东南片区拆迁70万平方米，“三网九园”建设初具雏形。扬州泰州国际机场一期扩建工程竣工启用，江广高速扩建工程建成通车，城南快速通道全线贯通。第六届中国-中亚合作论坛、2018年世界运河城市论坛、世界体育赛事与旅游峰会、第十届中国曲艺牡丹奖颁奖典礼、纪念朱自清诞辰120周年等重大活动成功举办。

生态环境明显优化。新增城市绿地158.5万平方米，新增植树665万株，林木覆盖率23.1%，自然湿地保护率51%，同比提升1.8个百分点。空气质量优良天数比率同比提升1.9个百分点。PM2.5年均浓度同比下降10.7%，降幅全省第一。完成5个集中式饮用水水源地整改任务，32个省考以上断面水质达标率93.8%。生态优势进一步彰显。

民生福祉持续改善。城乡居民人均可支配收入分别增长8.1%、8.7%。建档立卡低收入农户基本达到人均可支配收入7000元，脱贫率92.6%。全市高考每万人口普通类本二以上达线率蝉联全省第一。基层医疗卫生机构标准化率95%。新建城市书房10家。新建颐养示范社区22个。社会公众安全感达到98%，荣获全国创新社会治理示范城市。

两大盛会成功举办。坚持“办会为城、办会惠民、办成里程碑”理念，圆满承办第十九届省运会和第十届省园博会。新改建扬州体育公园、游泳健身中心、广陵体操馆等省运场馆20个，举办比赛6115场次，参赛人员2万人，扬州参赛总成绩创历史最好，在全省树立了“办会办赛的典范”。精心打造主展馆、民俗村等园博工程，高水准建成13个城市展园，吸引游客120万人次，实现了“一届比一届更精彩”的办会目标。

成绩来之不易，奋斗才有收获。面对宏观经济形势严峻、资源环境约束趋紧、社会稳定压力加大等诸多影响，我们迎难而上，积极作为，主要做了以下工作：

一是坚持发展第一要务，推动产业转型升级。强力推进产业项目招引和建设，新开工重大产业项目132个，同比增长6.5%。新落户世界500强及跨国公司项目6个。加快发展先进制造业，大力实施产业提质增效行动计划，规上工业增加值、开票销售、入库税收分别增长5%、15.7%、7%，净增规上工业企业130家。战略性新兴产业增加值占地区生产总值比重17%。扬州高新区获批国家高端装备制造业标准化试点。推进现代服务业提升规模，净增服务业重点企业121家，服务业增加值占比提高1个百分点；软件和信息服务业业务收入增长30%；金融业增加值增长4.5%，新增上市、挂牌企业5家，直接融资200亿元。入选全国旅游标准化试点城市，旅游业总收入900亿元，新增3A级以上景区8家。有效实施乡村振兴战略，新增设施农渔业12.3万亩，粮食总产量291.8万吨，新增地理标志商标5件。华东地区“中央厨房”及5个现代化生猪产业集聚区加快建设。

二是坚持改革创新引领，持续增强发展动能。紧紧围绕打造“新兴科创名城”，扎实推进“双创”基地城市示范建设。加大“放管服”改革力度，激发“双创”主体活力，推进行政审批服务“三减两免”，初步实现“3550”改革目标，全市“不见面”审批事项占比95.3%，全年降低企业税费负担143亿元。新增市场主体7.5万户，民间投资增长18%，全市小微企业营业收入同比增长9.2%。加快发展科创产业，新投用科技产业综合体65.7万平方米，新入驻企业1020家，新获批省级孵化器8家、众创空间14家；新签约产学研合作项目488个，引进研创中心42家，新建国家级博士后科研工作站4家，新增省级企业技术中心23家、工程研究中心11家；新获批国家技术创新示范企业2家、省级示范智能制造车间9家；新入选国家“千人计划”3人、“万人计划”5人、省“双创计划”领军人才（团队）33人。提高对外开放层次，全年实际利用外资12.2亿美元。实现出口83.5亿美元、外经营业额9.6亿美元，同比增长6%、5%。鼓励建筑业企业“走出去”，实现建筑业总产值3750亿元，增长6%。

三是坚持区域协调发展，提升城乡功能品质。构建立体交通格局。连淮扬镇高铁扬州段完成投资83%。沪陕高速枣林湾互通、352省道江都段、金湾路万福路以南段、观潮路跨古运河大桥建成通车，完成真州路与文昌路交叉口立体化改造。京沪高速扩容先导段、328国道仪征段、扬州西外环路、芒稻河特大桥和通扬线航道整治工程开工建设，启动快速路网二期建设。完善城市功能设施。瓜洲泵站完成水下主体工程，长江防洪能力提升一期工程全线开工。市区新建停车位2343个，新添置新能源公交车242辆，新辟调整公交线路26条，群众出行更为方便。加快推进“厕所革命”，全市新改建公厕53座。新铺设污水管网100公里。新建垃圾分类小区380个，通过国家餐厨废弃物资源化利用和无害化处置试点验收。开展环境综合整治。

实施“三路一环”“六路一环一河”整治提升工程，出新建筑立面57万平方米，改造亮化道路65公里、楼宇1400栋。建设美丽乡村，疏浚县乡河道65条、村庄河塘873条。新改建农村公路238公里、农桥438座。完成2个省级特色田园乡村和10个省级美丽乡村建设试点，11个重点中心镇全部创成国家级生态镇。

四是坚持绿色发展理念，用心守护蓝天碧水。开展“263”专项行动，坚决落实中央环保督察“回头看”整改任务。以壮士断腕的勇气，关停化工企业143家，整治黑臭水体52条111.7公里，完成153个行政村污水处理设施建设。秸秆禁烧实现“零火点、零通报”，主城区全面禁放烟花爆竹。发展低碳循环经济，实施重点用能单位“百千万”行动，单位地区生产总值能耗完成省定目标。新增绿色建筑标识面积129万平方米。推进生态廊道建设，加强长江生态大保护，取缔长江沿线非法码头22个。完成江淮生态大走廊建设项目23个，实施沿线乡镇污水处理厂提标改造及整合新建工程，高宝邵伯湖退渔还湖面积2.5万亩。

五是坚持发展为民导向，大力推动社会建设。推进高质量就业，城镇新增就业创业22万人次，城镇登记失业率控制在1.8%。完善社会保障体系，企业“五险”扩面新增4.6万人次，城乡居民医保保障待遇等“六统一”目标全面实现。提高教育均衡发展水平，新改扩建9所普惠性幼儿园，启动7所中小学建设，集中整治校外培训机构1109家。提升医疗卫生水平，农村区域性医疗卫生中心全面配建医疗急救站点，新创成二级医院6家，开工建设市公共卫生中心、妇女儿童医院，残疾人精准康复服务率98.6%，实现国家卫生城市“五连冠”。完善公共文化服务体系，开展公益文化活动470场，在全省率先实现公共文化机构全覆盖，“文明有礼24条”市民素质提升行动全面推开。推进社区颐养便民等设施建设，新建养老护理康复机构6个，护理型床位占养老机构床位总数47%；实施老小区加装电梯试点；建设社区邻里服务中心9个；改造提升市区农贸市场8个。深化平安扬州建设，圆满完成重大活动安保任务，扫黑除恶专项斗争成效明显，查处非法金融集资案47起；安全生产形势持续平稳。台湾事务、侨务、应急、民防、科普、档案、民族宗教、仲裁、妇女儿童、慈善、红十字等工作取得新进步。

六是坚持人民满意目标，加强政府自身建设。深入推进解放思想大讨论活动，努力营造高质量发展的浓厚氛围。制度化开展学纪学法，法治政府建设考评全省第一，“七五”普法中期考核通过验收。建成市县乡村政务服务“一张网”，建立网格化代办帮办服务体系，代办建设项目311个、帮办事项6427件。全年主动公开政府信息32万条，依申请公开892件。《寄语市长》、12345热线回复满意率分别达99%、99.6%。认真抓好巡视巡察反馈问题整改，积极化解政府隐性债务。自觉接受市人大及其常委会法律监督、工作监督和市政协民主监督，办理人大代表议案和建议313件、政协提案452件。落实党风廉政建设责任制，营造风清气正的政务环境。

各位代表，这些成绩的取得是中共扬州市委正确领导的结果，是市人大、市政协有效监督和大力支持的结果，是全市广大干部群众团结一心、努力奋斗的结果。在此，我代表市政府向全市人民，向市人大代表、政协委员，向各民主党派、工商联、无党派人士，向各人民团体、各界人士、人民警察、驻扬部队、驻扬单位，向所有关心支持参与扬州建设的海内外朋友们，表示崇高敬意和衷心感谢！

我们也清醒地看到，我市经济社会发展中还存在不少困难和问题，主要是：经济下行压力有所加大，实体经济发展面临较大困难；创新动能还不足，经济增长的内生动力需要进一步增强；环境治理任务还很艰巨，资源集约利用水平需要进一步提高；基本公共服务与群众期盼还有差距，城市精细化管理水平需要进一步提升；防范风险压力较大，互联网金融、中小企业“互保链”等方面的隐患需要进一步重视；有些干部担当精神不够，形式主义、官僚主义在一定程度上仍然存在。对此，我们将采取有力措施，切实加以解决。

2019年工作安排

2019年是新中国成立70周年，是决胜高水平全面建成小康社会的关键之年。政府工作总体要求是：以习近平新时代中国特色社会主义思想为指导，全面贯彻党的十九大、十九届二中、三中全会和中央经济工作会议精神，坚持稳中求进工作总基调，坚持新发展理念，坚持推动高质量发展，坚持以供给侧结构性改革为主线，统筹推进稳增长、促改革、调结构、惠民生、防风险工作，继续打好“三大攻坚战”，着力建设“三个名城”，全力办好“新十件大事”，推动“强富美高”新扬州建设取得新成效，为高水平全面建成小康社会打下决定性基础。

按照市委七届七次全会总体部署要求，综合各方面因素，本着担当作为、求真务实的工作导向，今年主要预期目标是：地区生产总值增长7%左右，一般公共预算收入增长5.5%以上，固定资产投资增长9%以上，制造业投资占比60%，社会消费品零售总额增长8.5%以上，全社会研发投入占地区生产总值比重2.55%，居民人均可支配收入增长8%左右，城镇登记失业率控制在4%以内，居民消费价格上涨不高于省定目标，单位地区生产总值能耗和大气、水环境质量确保完成省定目标。

围绕实现上述目标，重点抓好以下八个方面工作：

一、聚焦实体经济，不断夯实高质量发展根基

持续壮大先进制造业。实体经济是硬实力，先进制造业是硬支撑，是我市“三个名城”建设的家底子。紧扣创新型、全产业链、可持续发展方向，打造一批“拆不散、搬不走、压不垮”的产业集群。推动三大千亿级优势产业提升竞争力、迈向中高端。汽车产业，重点发展整车、新能源汽车、智能网联汽车及关键零部件，全力推进上汽大众仪征二期项目建设。高端装备产业，突出智能制造方向，发展高档数控机床、工程及精密液压、工业机器人等行业。新型电力装备产业，重点提升扬州智能电网产业基地、高邮国家火炬特种电缆产业基地发展水平，推进宝应光伏发电“领跑者”基地建设。推动一批百亿级传统产业壮大规模、转型升级。船舶制造产业，加快向海工装备、特种专用船舶、船舶电子方向提升。高端纺织和传统服装产业，主攻纺织纤维新材料、品牌服装家纺等领域，提升品牌效应和市场份额。食品行业，加快构建研发、加工、物流、销售全产业链，推进华东地区“中央厨房”建设。推动战略性新兴产业紧盯产业前沿、加快创新发展。新能源新光源产业，做大太阳能光伏发电和半导体照明应用规模，扩大太阳能产业集聚优势。生物医药产业，重点发展新医药、生物医学工程和新型医疗器械，推动扬州高新区生物健康产业园、广陵区健康医疗产业园做大规模、提升质态。微机电产业，深化与清华大学合作，加快建设微机电产业园和研究院。推动各项产业政策集成创新、协同发力。加快实施培育先进制造业集群三年行动计划和创新驱动、智能制造等“六大工程”。大力发展军民融合产业，积极争创省级军民融合示范区。全市规上工业增加值、开票销售分别增长7%、8%以上。高新技术产业产值占规上工业产值比重47%，战略性新兴产业增加值占地区生产总值比重达到17.5%。

加快“园区经济”转型升级。园区兴则产业兴，园区强则经济强。在特色发展上求突破，明确园区产业主攻方向，培育壮大龙头企业，形成特色产业集群，每个省级开发园区培育发展产销规模50亿元以上龙头企业2家以上。在集约发展上重实效，完善企业资源集约利用综合评价体系，加大对闲置、低效和批而未用土地的处置力度，鼓励企业建设多层厂房。省级以上开发园区亩均开票销售、亩均入库税收分别达200万元、14万元。在服务功能上抓提升，优化政务、商务、财务、法务和投融资服务，强化核心区与生活区、商务区、办公区等功能配套衔接。在争先创优上促进位，市开发区、扬州高新区力争进入全省同类园区前10位，江都、维扬开发区进入全省前15位，仪征开发区进入全省前20位，宝应、高邮开发区前进5位。全年省级以上开发园区地区生产总值、一般公共预算收入、实际利用外资、自营出口分别占全市58%、38%、72%、80%以上，园区经济对经济增长贡献率提高5个百分点。

促进项目建设提质增效。抓项目就是抓发展、抓未来。更大力度招引大项目好项目，新引进重大产业项目240个以上，推动100亿元、50亿元重特大产业项目和成长性强的科创项目取得新突破。更高效率推进重大项目建设，每月召开重大项目过堂会，建立任务倒逼、时序倒排、责任倒查机制，健全“签约注册、到资开工、投产达效、纳税就业”全流程工作链条，提高项目竣工率、达产率。更严要求提高项目运营质态，出台更刚性的考核办法，加大对重大项目亩均投资、亩均开票、亩均税收和竣工后研发投入等关键指标的考核评价。

二、强化创新驱动，全力建设“新兴科创名城”

积极发展“城区经济”。科创名城是主航道，“城区经济”是主动能。提升“三室经济”发展水平，科技研发产业，全力引进代表国家水平的科研院所和实验室在扬设立研究机构，打造科创名城的金色名片；重点围绕先进制造、现代农业、生物健康、新材料等领域，加快共建中航沈阳飞机研究所协同创新研究院等一批国家级、省部级重点实验室。软件和信息服务业，聚焦核心工业软件、应用软件等关键技术，加快推进腾讯云大数据中心等重大项目，着力培育50个具有自主知识产权的软件产品。文化创意产业，加快推动创意设计、工艺美术、体育赛事、文博会展等行业发展，着力培育和引进文化体育骨干企业。提升总部经济集聚水平，引进和推动大企业、大集团在扬州设立区域研发中心、营销中心、结算中心等功能性总部；鼓励本土企业总部立足本地、拓展全球、多做贡献。提升科技产业综合体建管用水平，全年新开工科技产业综合体100万平方米，累计投入使用率75%，新增省级以上孵化器、众创空间10家，新引进企业1000家、人才1万人以上，小微企业“双创”基地城市示范建设通过国家验收。全年净增服务业重点企业100家，服务业增加值占地区生产总值比重提高1个百分点。

持续推动技术创新。以自主创新为引领，推动产业链与创新链相融合。突出企业创新主体地位，培育省级以上企业技术中心100家，实施重点技术创新项目100项，净增国家高新技术企业150家，新增国家科技型中小企业500家；万人发明专利拥有量超15件，新增注册商标5000件。突出公共服务平台支撑作用，加快工业互联网等公共服务平台建设，培育引进科技服务机构20家，技术产权交易合同备案登记额超11亿元。突出产学研协同创新，实施关键共性技术攻关和重大科技成果转化各100项，达成产学研合作协议400项，新增省级以上“两站三中心”30家以上。

加速集聚创新要素。加大人才资源引培力度，深入实施“绿扬金凤计划”，力争引进创新创业领军人才（团队）100人、优秀博士100人、科技副总100人。推进扬子津科教园区建设，完成南邮通达学院二期工程，推进江苏旅游职业学院配套工程建设，扬州人力资源服务产业园争创省级园区，新增在扬就业创业大学生1万名以上。开工建设扬州技师学院新校区，研究规划建设护理学院和幼儿师范学院。实施专业技能培训10万人次，每万劳动力中高技能人才880人。加大金融支持力度，设立先进制造业贷款资金池，“苏科贷”“扬科贷”等各类风险池规模增至20亿元；充分发挥政府投资基金对创新型企业的撬动作用，全年发放创新券、技改券、服务券各1亿元，全市政府性投资基金规模100亿元以上。

三、深化改革开放，充分激发内生增长动力

扎实推进重点领域改革。聚焦供给侧结构性改革，严格落实“巩固、增强、提升、畅通”的工作要求，严控化工、钢铁等行业过剩产能；防范和化解政府隐性债务，推动融资平台公司市场化转型；全面落实减税降费政策，全年降低企业负担100亿元以上；加快实施“补短板专项工程”。持续深化“放管服”改革，巩固扩展“3550”成效，推进“不见面”审批服务便民化改革，全面推行“证照分离”，进一步提升“互联网+政务服务”水平，实现企业、群众办事更方便、更快捷。统筹推进市属国有企业、农村集体产权制度、经济发达镇行政管理体制等领域改革。做好第四次经济普查工作。

大力支持民营经济发展。民营经济发展空间巨大，民营企业家舞台广阔。进一步优化民营经济发展环境，制定实施民营经济高质量发展意见，在市场准入、审批许可、招投标、政府采购等领域破除障碍、降低门槛，积极促进投资便利化，全年新登记市场主体5万户。进一步支持民营企业做强做优，加大政策性融资担保力度，切实解决好民营企业融资难、融资贵问题。鼓励企业利用资本市场做大做强，动态培育重点上市后备企业30家，力争新增上市、挂牌企业5家，企业直接融资200亿元以上。进一步提振民营企业家发展信心，切实帮助民营企业解决因政策变化而产生的历史遗留问题，让民营企业家丢掉包袱、轻装上阵。

着力提升对外合作水平。积极对接“一带一路”、长江经济带、长三角一体化和宁镇扬一体化等国家和省重大战略，抢抓机遇，有效作为。加快跨江融合发展，强化产业合作，深化与国家级南京江北新区对接，打造毗邻地区发展示范区；推进交通、公共服务一体化，在过江通道、城轨互通、生态环保、文化旅游、市民服务等方面共建共享，取得实质性进展。促进利用外资提质增效，全年新引进世界500强及跨国公司项目6个、外资并购项目2个，实际利用外资13亿美元。促进对外贸易优进优出，出台新一轮外贸稳增长政策，落实贸易便利化措施，大幅降低进出口环节合规成本，全年出口85亿美元，一般贸易占货物进出口总额比重稳定在70%以上。促进企业拓展境外市场，支持中阿（联酋）产能合作示范园、缅甸农产品加工园等境外经贸合作平台建设，鼓励企业开拓海外市场，实现对外投资总额5亿美元。引导建筑业企业参与“一带一路”沿线国家基础设施项目建设，全市建筑业总产值突破4000亿元。

四、完善功能品质，精心打造美丽宜居“公园城市”

做优做美中心城市。高水平做好城市总规修编，高质量推进城市“双修”。聚焦江广融合区发展，加快推进“三河六岸”公园、高铁枢纽、龙川之窗等一批重点项目，打造优美生态与高端产业相融合的城市新地标。加快东南片区更新改造，突出三湾湿地公园功能提升，实施七大类56个项目，为实现“三年定局、五年完成”目标打好基础。加强古城保护和利用，启动实施三和四美地块历史文化街区整治及修复、仁丰里二期、四望亭广场、文昌阁修缮等工程，完成南通路-南河下街区综合整治。推进公园体系建设，贯彻落实《扬州市公园条例》，新建滨江公园等各类公园28个，做好62个公园的绿化补植和9个公园的服务设施增补工作。高标准规划建设世博园。推进“垃圾革命”，加强垃圾分类集中处理，分类投放设施覆盖率60%；提升改造老城区垃圾池38个，建成餐厨废弃物处理设施3座、建筑垃圾处理厂2座、再生资源可回收物分拣中心1座，市区生活垃圾焚烧发电厂三期、赵庄生活垃圾卫生填埋场二期项目主体竣工。

提升城市服务功能。完善城市路网体系，加快连淮扬镇高铁扬州段建设，促进北沿江高铁扬州段尽快开工；开工建设龙潭过江通道；加快润扬第二过江通道、宁扬、宁马城际等项目前期工作；推进328国道仪征段改扩建，加快扬州西外环路、333省道高邮东段、331省道宝应段建设，实施京沪高速公路扬州段改扩建工程；加快万福路、江平路、润扬路、运河南北路等城市快速路网建设；金湾路建成通车，完成文峰路南延、连运路拓宽改造、扬子江北路提升改造、施井路整治工程。综合采取节点改造、平交路口渠化、瓶颈路段加宽等措施，使道路通行更方便。完善城市防洪体系，瓜洲泵站主体工程全面建成；完成长江防洪能力提升一期工程、启动二期工程，开工建设乌塔沟综合整治工程。完善城市公用设施体系，启动“中央水库”规划建设；实施第四水厂、头桥水厂深度处理改造；推动液化天然气应急调峰储备站建设；推进城市地下管线地理信息共享专线建设。启动北山污水处理厂建设，做好雨污分流及污水管网的检测和疏通整治。全年新改建城市公厕103座。

强化精致精细管理。认真落实文明城市建设常态化长效化三年行动计划，聚焦重点部位、薄弱区域，继续开展突出问题综合整治，巩固文明城市创建成果。加快构建“大城管”体制，下沉城管执法力量，做实基层管理网格。深化“云上扬州”建设，提升城市智能化管理水平。实施城市安全发展行动计划，着力推进城市应急管理、隧道高架救援救助等工作，建成投用市高层建筑专业救援消防站。

五、聚力乡村振兴，促进“强农惠农富农”取得新成效

加快推进农业现代化。加大“三农”投入力度，积极引导社会资本更多更好参与乡村振兴。现代农业重点实施“四大工程”：粮油绿色增效工程，落实粮食安全责任制，新建高标准农田15万亩；设施农业“机器换人”工程，推广应用现代化智能化设施装备，新增高效设施农渔业8万亩；优势品牌提升工程，全年新增绿色、有机农产品30个，新创地理标志商标2件，绿色优质农产品比重40%；农业产业化发展工程，培育新型农业经营主体，新增省级农业产业化联合体7家，新上“农业板”挂牌企业8家，全年新竣工农业重大项目20个。

全面提升乡村建设水平。高质量推进“四好农村路”建设，加强县道、乡道以及通公交村道安全隐患治理，行政村双车道四级公路覆盖率90%以上，打造“四好农村路”示范乡镇6个。高标准开展农村人居环境整治，疏浚县乡河道50条，整治村庄河塘200条，打造美丽宜居乡村150个。学习借鉴浙江“千万工程”经验，开展农民集中居住区试点规划建设。全面启动被撤并乡镇综合整治，改造农村危房1033户，建设村庄污水处理设施120个；新创10个省级水美村庄。高要求完善乡村公共服务设施，在全市农村地区全面建立公办幼儿园和普惠性民办幼儿园服务区制度，规划建设一批便民服务中心、便民超市、卫生服务机构，乡镇卫生院、村卫生室示范化建设比例高于省均5个百分点以上。

不断拓宽脱贫致富路径。强化精准扶贫、精准脱贫，落实“三保五助”政策，全市建档立卡低收入农户人均可支配收入稳定达到7000元，实现全面脱贫目标。建立健全职业农民培育机制，全年培育新型职业农民1.3万人。鼓励发展新产业新业态，全市休闲观光农业年接待游客650万人次，营业收入增长20%以上；农业电商年销售额达65亿元。健全“阳光三农”监管系统，加强农村集体“三资”监管。支持发展农村集体经济，村级集体经济增收5%以上、村级债务下降5%以上，沿河、沿江地区所有市级经济薄弱村集体经济收入分别达45万元、55万元。

六、坚守绿色发展，着力推动生态文明建设走在前列

持续打好污染防治攻坚战。坚持全民共治，抓好中央环保督察“回头看”反馈问题整改落实。坚决打赢蓝天保卫战，开展清洁柴油车、清洁油品等四大攻坚行动，市区新购清洁能源公交车254辆，深度治理工业大气污染，严格管控各类扬尘，PM2.5年均浓度同比下降2%，继续走在全省前列。着力打好碧水保卫战，深化“河长制”“湖长制”，整治集中式饮用水水源地保护区内违法违规问题，各县（市、区）全部建成应急水源；加大船舶污染管控力度；加快城镇污水收集和处理设施建设，全面完成城市建成区黑臭水体整治任务。扎实推进净土保卫战，开展重点行业企业用地调查，强化联动监管，有序开展污染地块修复治理和风险防控。

积极推动绿色生产。严格落实江淮生态经济区建设“四个清单”，坚决守住生态保护红线。大力推广循环经济，全面实施市级以上园区循环化改造，加快推动国家循环经济示范城市建设；落实48家重点用能企业“双控”目标，实施100项节能改造项目，实现节能10万吨标准煤。鼓励新能源和可再生能源项目建设，开展绿色园区、绿色工厂、绿色产品、绿色供应链示范创建工作，积极推进省级生态工业园区建设。加大化工企业“四个一批”推进力度，实现扬农集团和联环集团老厂区全面停产。

加快生态廊道建设。构建生态廊道和生物多样性保护网络，呈现“绿杨城郭”“鸟飞蛙鸣蝉唱”的生态画卷。实施“一园两廊三片四沿多点”重点生态工程，新增成片造林4.2万亩，全市林木覆盖率23.3%以上。修复湿地4000亩，新建湿地保护小区3个。坚决打好长江保护修复攻坚战，大幅提升生态岸线比例，干流及洲岛岸线开发利用率降到50%以下，消除主要入江支流劣V类水质。持续推进江淮生态大走廊建设，高宝邵伯湖退渔还湖面积1万亩，全面实现退养还湖，主要入湖河流水质稳定达标。做好南水北调清水廊道1公里范围内植树造林工作，确保一江清水向北送。

七、加快文旅融合，深入推进“国际文化旅游名城”建设

完善公共文化服务体系。提高群众文化活动参与度，实施全民艺术普及行动计划，开展公益文化活动200场次以上；举办庆祝新中国成立70周年系列活动，承办第五届金狮奖全国木偶皮影展演。争创全民运动健身模范市，举办好第13届市运动会和鉴真国际半程马拉松赛。提高文化资源覆盖面，新建城市书房10个，加快建设新大剧院、广陵公共文化中心；组建县级融媒体中心，建成乡镇健康促进服务中心6个，村级综合文化服务中心实现全覆盖，人均公共文体设施面积达2.81平方米。提高传统文化生命力，贯彻落实《扬州市非物质文化遗产保护条例》，开展第三批国家级非遗代表性传承人抢救性记录工作；加强名师大家和高水平创作人才培养，打造一批精品力作。

发展壮大“景区经济”。发展全域旅游，开发市县旅游精品线路，构建旅游一体化格局；提升县域景区品质和数量，推动县（市、区）实现4A级景区全覆盖。发展全业旅游，推动旅游与会展、赛事等产业融合发展，开发互动参与、红色经典等旅游产品，加快建设华侨城、江都光线中国电影世界等标志性文化旅游项目。发展全时旅游，打造四季旅游品牌，提升过夜游客占比；积极做好“景区+”文章，布局建设一批酒店群。加快推进国家旅游标准化示范城市建设，旅游业总收入突破千亿元。

高水平推进大运河文化带建设。打造运河文化展示窗口，启动建设中国大运河博物馆，使其成为大运河沿线国家文化公园建设最重要的标志，推进扬州城国家考古遗址公园、大运河国际非遗文博园、隋炀帝墓考古遗址公园等文博园区建设，加快形成“一馆多园”格局。举办中国大运河申遗成功5周年纪念活动，承办好省首届大运河文化旅游博览会、世界运河城市论坛暨世界运河大会。打造运河文化保护典范，加强大运河扬州段遗产保护、传承和利用，重点推进宝应刘堡闸、高邮明清运河故道、运河邵伯古镇段遗产、淮扬运河主线广陵段保护及环境整治工程。打造运河文化产业高地，促进玉雕、古琴、刺绣等非遗资源活化转化，486非遗集聚区、湾头玉文化产业园、甘泉琴筝文化产业园、鲁垛乱针绣产业园等争创省级文化产业示范园区。

八、顺应民生需求，更好满足人民群众新期盼

提高就业质量和社会保障水平。推动就业惠民，注重解决结构性就业矛盾，构建“互联网+”就业创业公共服务体系，城镇新增就业5.5万人，援助困难人员再就业5000人以上。推动社保利民，全面推进企业职工基本养老保险市区统筹，实现企业“五险”扩面新增2.5万人次以上。推动救助安民，落实低保标准城乡一体化自然增长机制，完善残疾儿童康复救助制度，促进慈善等社会救助与扶贫开发、医疗救助与大病保险有效衔接。

推进基本公共服务均等化。坚持学有优教。新改扩建7所普惠性幼儿园，加强民办幼儿园监管，加快解决儿童入园难问题；启动建设4所中小学，高中教育优质学校比例达78%。坚持病有良医。完善分级诊疗制度，推进紧密型医联体建设，4家区域性医疗卫生中心创成二级医院；加快妇女儿童医院建设，完成市传染病院改扩建工程，市公共卫生中心主体封顶。坚持老有善养。推动农村敬老院提档升级，新建颐养示范社区23个；实施市区职工长期护理保险制度试点，优先满足重度失能老人养老医疗服务需求，护理型养老床位占比提升至51%，养老机构医疗服务基本全覆盖。

加强和创新社会治理。持续提升基层治理水平，扎实开展新时代文明实践中心建设试点工作，有序推进网格化精细化社会治理工作，整合下沉网格力量，确保网格化创建达标率80%以上。深化平安扬州建设，推动公安大数据中心建设，集成公安、城管、社保、民政和12345等系统，加快构建社会治理数据共享应用平台；深入开展扫黑除恶专项斗争，社会公众安全感继续走在全省前列。持续提升矛盾化解水平，深入推进领导干部接访下访和包案化解信访突出问题，健全人民调解、行政调解、司法调解联动机制，把矛盾化解在源头，让和谐形成氛围。持续提升安全保障水平，落实安全生产责任制，突出抓好安全隐患整治，提高应急管理能力和防灾减灾水平。加强食品药品安全监管。防范和化解各类金融债务风险，加大非法集资、通讯网络诈骗等违法犯罪活动的打击力度。加强国防动员建设，做好退役军人事务和双拥共建工作。

全面实施2019年民生“1号文件”，全力以赴办好重点民生实事，确保实现向人民群众作出的庄严承诺。

全面加强政府自身建设

各位代表，新时代、新征程，对政府工作提出了更高的要求。我们一定不忘初心，牢记使命，不断提高政府治理能力，努力建设人民满意的政府。

加强政治建设。旗帜鲜明讲政治，增强“四个意识”，坚定“四个自信”，坚决维护习近平总书记的核心地位和党中央集中统一领导，自觉用习近平新时代中国特色社会主义思想武装头脑、指导实践、推动工作。坚持把党对一切工作的领导贯彻到政府工作各个领域各个方面，确保中央大政方针、省委省政府决策部署和市委工作要求落地生根。

坚持依法行政。深化法治政府建设，自觉运用法治思维和法治方式推进工作。加强对行政权力的制约和监督，依法接受人大及其常委会的监督，主动接受人民政协的民主监督，接受社会和舆论监督。加强政府内部层级监督，完善政府规章制度，扎紧制度笼子，让权力在阳光下运行。把诚信施政作为重要准则，说到做到、一诺千金，建设诚信政府。

增强履职本领。硬肩膀才能挑起重担子。新时代担当新使命，既要政治过硬，也要本领高强、勇于担当。深入推动学习型政府建设，打牢全面、系统、专业的知识根底，提高适应新时代、落实新要求的专业化能力。直面矛盾问题，做到敢扛事、会做事、做成事。按照“五可”要求推进任务项目化、项目清单化、清单责任化，切实增强政府执行力。

强化勤政为民。坚持以人民为中心的发展思想，始终把老百姓的烦心事、揪心事和安危冷暖放在心上，用心用情用劲为老百姓做事。多到困难多、矛盾大的地方去，把功夫下在摸实情、出实招、办实事上，埋头苦干不张扬，久久为功求实效。

打造廉洁政府。深入贯彻落实全面从严治党和党风廉政建设各项规定要求，加强源头治理，用制度管权管事管人。加大审计监督力度，严管公共资金，严管公共资源交易，严管国有资产，树立政府“过紧日子”的思想，把有限的资源和财力用在推动发展、改善民生上。严守廉洁从政各项规定，守住底线，不踩红线，永葆为民务实清廉的政治本色。

各位代表，实干收获希望，奋斗创造未来。让我们紧密团结在以习近平同志为核心的党中央周围，以习近平新时代中国特色社会主义思想为指导，在中共扬州市委的坚强领导下，加满油，鼓足劲，一起拼搏、一起苦干、一起奋斗，把“人们心目中的扬州”建设好，谱写“强富美高”新篇章，以高质量发展的新业绩向新中国成立70周年献礼！
